# Low-Energy Mechanism Causing an Unusual Presentation of a Femur Fracture

**DOI:** 10.1016/j.acepjo.2025.100099

**Published:** 2025-03-13

**Authors:** Kahra Nix, James Webb, Luther Daniel, Matt Eisenstat

**Affiliations:** Department of Emergency Medicine, University of Louisville, School of Medicine, Louisville, Kentucky, USA

**Keywords:** geriatric patient, low-energy mechanism, femur, femoral shaft fracture, closed reduction, hospice

## 1 Patient Presentation

**Figure 1:**
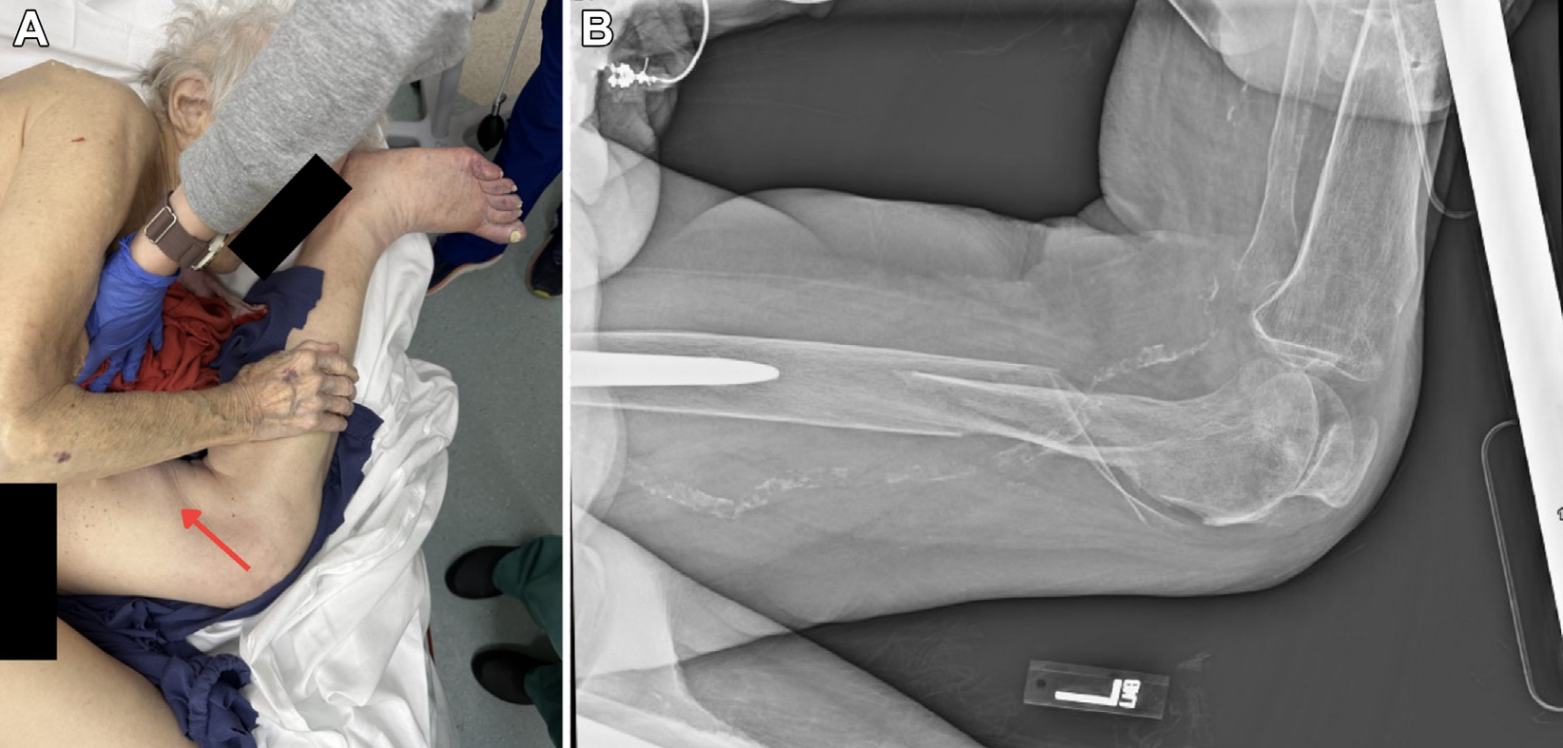
(A) A fall from the bed left the patient with rolled left lateral decubitus, and the left hip flexed with rotation at a mid-thigh deformity (red arrow) so that the lower leg was cephalad. A single-view lateral radiograph of the left femur (B) showed a laterally displaced, comminuted femoral shaft fracture.

A 99-year-old patient presented after a ground-level fall. She was transferred to the stretcher as she was found at her facility in the left lateral decubitus position. She denied having a head or neck strike. She was alert, oriented, and calm. The left hip was flexed, and there was rotation around a mid-thigh deformity so that the lower leg was cephalad (Fig 1A). The skin and neurovascular exams were intact.

## 2 Diagnosis: Femoral Shaft Fracture

**Figure 2:**
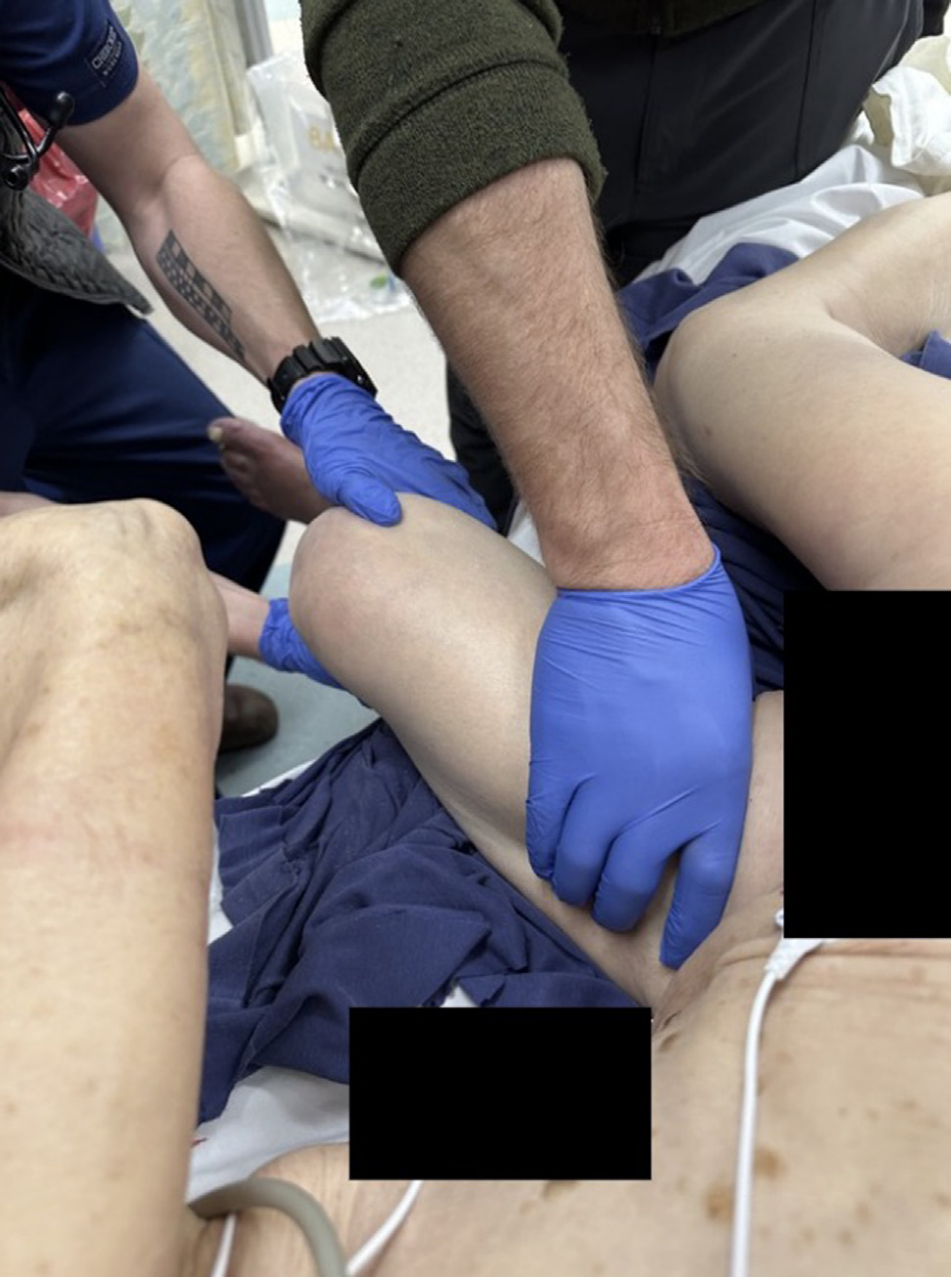
Repositioning of the patient on the emergency department stretcher to allow for reduction around the mid-thigh defect.

**Figure 3:**
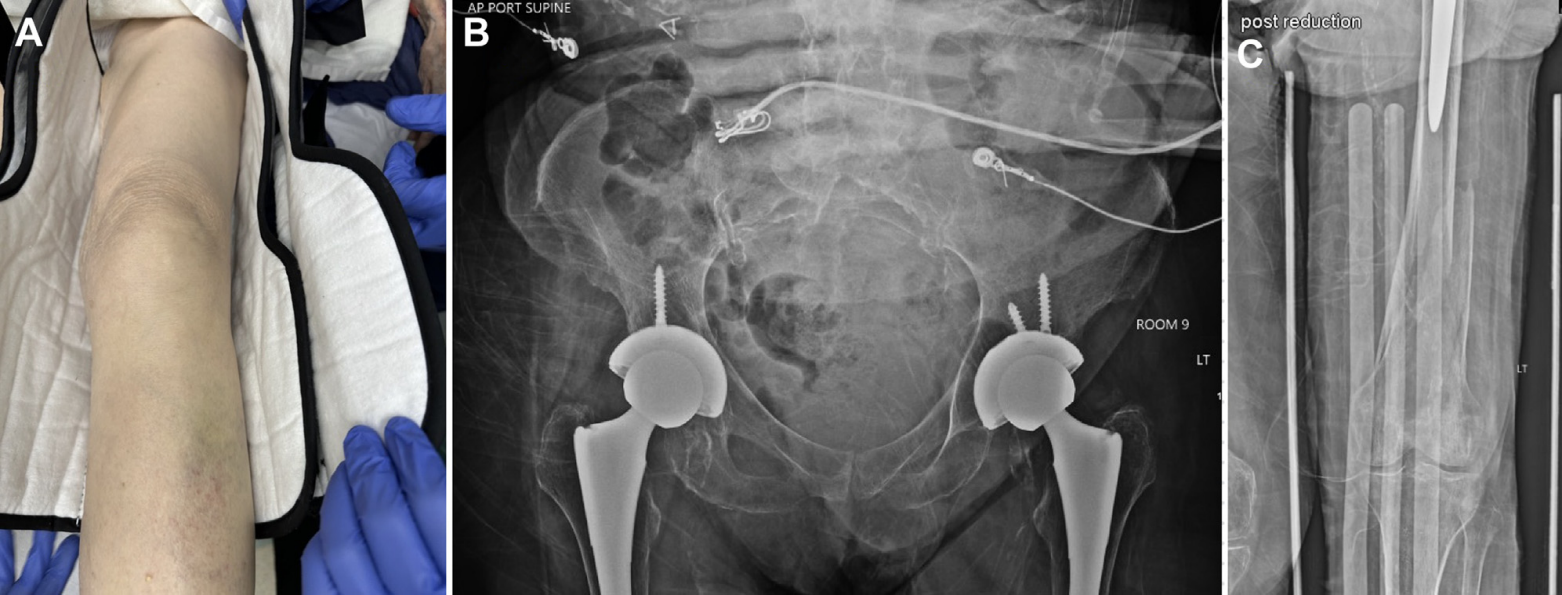
(A) The previously deformed and rotated left leg is now positioned in anatomic alignment. Single-view, postreduction, anteroposterior radiographs of the pelvis (B) and femur (C) showed bilateral total hip arthroplasty with acetabular screws but no loosening, fracture, or dislocation and the femoral shaft fracture.

A single-view lateral radiograph of the left femur showed a laterally displaced, comminuted femoral shaft fracture (Fig 1B). We moved her while maintaining her position until her leg was no longer on the stretcher; this allowed us to rotate 180 degrees at the defect in the left mid-thigh (Fig 2). The leg was pulled to length and placed in a knee immobilizer with a plan for traction (Fig 3A). Postreduction anteroposterior radiographs of the pelvis (Fig 3B) and left femur (Fig 3C) showed bilateral total hip arthroplasty with acetabular screws but no loosening, fracture, or dislocation and the previously seen femoral shaft fracture. Her pain was controlled with oral and intravenous opioids. Computed tomography angiography of the left leg did not demonstrate any additional injury. Femoral shaft fractures are often associated with high-energy trauma, and it is common to find additional associated injuries.^1^ Her metastatic breast cancer and multiple comorbidities under hospice care likely explain how this injury resulted from a low-energy mechanism. Orthopedics offered operative treatment or immobilization with outpatient hospice. She chose hospice care and died 48 hours later.

## Funding and Support

Not applicable.

## Conflict of Interest

All authors have affirmed they have no conflicts of interest to declare.
